# Glucometabolism in Kidney Transplant Recipients with and without Posttransplant Diabetes: Focus on Beta-Cell Function

**DOI:** 10.3390/biomedicines12020317

**Published:** 2024-01-30

**Authors:** Amelie Kurnikowski, Benedetta Salvatori, Michael Krebs, Klemens Budde, Kathrin Eller, Julio Pascual, Micaela Morettini, Christian Göbl, Manfred Hecking, Andrea Tura

**Affiliations:** 1 Department of Epidemiology, Center for Public Health, Medical University of Vienna, 1090 Vienna, Austria; 2 CNR Institute of Neuroscience, 35127 Padova, Italy; benedetta.salvatori@in.cnr.it (B.S.); andrea.tura@cnr.it (A.T.); 3 Division of Endocrinology and Metabolism, Department of Internal Medicine III, Medical University of Vienna, 1090 Vienna, Austria; 4 Medizinische Klinik m. S. Nephrologie, Charité Universitätsmedizin Berlin, 10117 Berlin, Germany; 5 Clinical Division of Nephrology, Department of Internal Medicine, Medical University of Graz, 8036 Graz, Austria; 6 Institut Hospital del Mar d’Investigacions Mèdiques (IMIM), 08003 Barcelona, Spain; 7 Department of Nephrology, Hospital Universitario 12 de Octubre, 28041 Madrid, Spain; 8 Department of Information Engineering, Università Politecnica delle Marche, 60131 Ancona, Italy; m.morettini@univpm.it; 9 Department of Obstetrics and Gynaecology, Medical University of Graz, 8036 Graz, Austria; christian.goebl@meduniwien.ac.at; 10 Division of Nephrology and Dialysis, Department of Internal Medicine III, Medical University of Vienna, 1090 Vienna, Austria; 11 Kuratorium for Dialysis and Kidney Transplantation (KfH) e.V., 63263 Neu-Isenburg, Germany

**Keywords:** PTDM, NODAT, beta-cell function, insulin resistance, pancreatic alpha-cell, kidney transplantation, endocrine pancreas

## Abstract

Posttransplant diabetes mellitus (PTDM) is a common complication after kidney transplantation. Pathophysiologically, whether beta-cell dysfunction rather than insulin resistance may be the predominant defect in PTDM has been a matter of debate. The aim of the present analysis was to compare glucometabolism in kidney transplant recipients with and without PTDM. To this aim, we included 191 patients from a randomized controlled trial who underwent oral glucose tolerance tests (OGTTs) 6 months after transplantation. We derived several basic indices of beta-cell function and insulin resistance as well as variables from mathematical modeling for a more robust beta-cell function assessment. Mean ± standard deviation of the insulin sensitivity parameter PREDIM was 3.65 ± 1.68 in PTDM versus 5.46 ± 2.57 in NON-PTDM. Model-based glucose sensitivity (indicator of beta-cell function) was 68.44 ± 57.82 pmol∙min^−1^∙m^−2^∙mM^−1^ in PTDM versus 143.73 ± 112.91 pmol∙min^−1^∙m^−2^∙mM^−1^ in NON-PTDM, respectively. Both basic indices and model-based parameters of beta-cell function were more than 50% lower in patients with PTDM, indicating severe beta-cell impairment. Nonetheless, some defects in insulin sensitivity were also present, although less marked. We conclude that in PTDM, the prominent defect appears to be beta-cell dysfunction. From a pathophysiological point of view, patients at high risk for developing PTDM may benefit from intensive treatment of hyperglycemia over the insulin secretion axis.

## 1. Introduction

Posttransplant diabetes mellitus (PTDM) is a common complication affecting a remarkable proportion of kidney transplant recipients, with a high risk for cardiovascular events and mortality [1,2,3,4,5]. Immediately after kidney transplantation, up to 90% of all patients experience hyperglycemic episodes, mainly as a consequence of high steroid and calcineurin inhibitor doses [6]. This vulnerable early phase may play a pivotal role in the later development of PTDM [7]. The incidence of PTDM has been reported to vary from approximately 10% to 40% in the first year after transplantation [8,9]. Clinical risk factors such as central obesity, lack of physical activity, sedentary lifestyle, and viral infections (e.g., hepatitis C virus and cytomegalovirus) have been reported, in addition to the diabetogenic effects of the immunosuppressive agents used in posttransplant therapy [8,10,11,12,13,14]. The mechanisms leading to PTDM also include early postoperative stress and increased insulin demand due to restoration of kidney function and, hence, augmentation of insulin clearance [15].

PTDM may display some pathophysiological differences compared to type 2 diabetes (T2DM). In T2DM, two main processes typically contribute to the development of the disease, i.e., impairment in insulin sensitivity and in beta-cell function, the former being the ability of insulin to promote glucose disposal, and the latter being the ability of the beta cells to release insulin in response to changes in plasma glucose concentration. In PTDM, controversy about the pathophysiologic mechanisms persists [16,17]. Earlier studies have found that beta-cell dysfunction appears to be the predominant defect rather than an impairment in insulin sensitivity (insulin resistance) [18,19]. In our own previous analysis, we found that insulin sensitivity at similar 2 h glucose concentration values during an oral glucose tolerance test (OGTT) was even higher in kidney transplant recipients than in nontransplant control subjects from the general population, whereas insulin release was lower [20]. Based on these premises, it sounds conceivable that early treatment of kidney transplant recipients with insulin administration may have beneficial effects in terms of PTDM prevention. Using the framework of randomized controlled clinical trials, we assessed whether postoperative hyperglycemia in kidney transplant recipients without previous diabetes diagnosis could be controlled using basal insulin therapy. Our strategy in the intervention group was to treat with basal insulin according to afternoon glucose levels, which are generally higher than fasting if glucocorticoids are administered in the morning [21]. Our findings showed that the described intervention based on early basal insulin therapy following kidney transplantation reduced the odds for PTDM at 12 and 24 months posttransplant in subjects that adhered to the treatment protocol [22,23].

Despite this treatment approach, however, some kidney transplant recipients develop PTDM. In view of the argument surrounding the pathophysiology of PTDM, we aimed to use OGTT-derived data to compare the glucometabolism of kidney transplant recipients who develop PTDM in comparison to that of patients who remained diabetes-free. Specifically, we compared kidney transplant recipients with and without PTDM at 6 months after transplantation, placing the focus on beta-cell function and insulin sensitivity, as assessed by both basic indices and model-based parameters.

## 2. Materials and Methods

### 2.1. Patients and Experimental Procedure

The present analysis was predefined in the protocol of our multicenter randomized controlled trial “Insulin Therapy for the Prevention of New Onset Diabetes After Transplantation” (ITP-NODAT, ClinicalTrials.gov NCT03507829). The ITP-NODAT study was undertaken from November 2012 through May 2018 at four European transplant centers (Medical University of Vienna, Austria; Medical University of Graz, Austria; Hospital del Mar Barcelona, Spain; Charité Universitätsmedizin Berlin, Germany). The study was conducted with independent external monitoring in accordance with ICH-GCP principles and the Declaration of Helsinki. Written informed consent was obtained from all patients following approval from the institutional review board at each participating center.

The ITP-NODAT study included 263 adult kidney transplant recipients without a history of pretransplant diabetes and receiving glucocorticoids, mycophenolate acid, and tacrolimus. They were randomized 1:1 to daily postoperative four-point capillary glucose measurements and Neutral-Protamin-Hagedorn (NPH) insulin intervention for afternoon glucose ≥140 mg/dL versus standard-of-care for the prevention of PTDM. For more detailed information, please refer to our previous articles on our earlier proof-of-principle study [22], on the clinical outcome (including primary endpoint) of ITP-NODAT [23] and on the diagnostic criteria for PTDM and prediabetes [24]. After the baseline visit immediately pre-transplantation, follow-up included an OGTT at 6 months (double-blinded), 12 months and 24 months posttransplant. Participants exited the study either by completion at 24 months or before due to death, graft loss or withdrawal of consent.

For the OGTT, participants ingested 75 g of glucose dissolved in water after an 8 h overnight fast. Plasma and serum samples were collected at fasting state (0 min) and after 30, 60, 90 and 120 min for measurement of glucose, insulin and C-peptide. PTDM was defined according to the American Diabetes Association criteria for diabetes mellitus [25]. In line with the ITP-NODAT study, participants were classified as having PTDM based on OGTT-derived 2 h plasma glucose or by use of glucose-lowering medication. The focus of the current analysis was on participants diagnosed at 6 months posttransplant. In the present analysis, we selected those patients having at least 4 out of the 5 samples at the 6 months OGTT (4 samples for every OGTT variable, i.e., glucose, insulin and C-peptide). In addition to the OGTT data, several other anthropometric, sociodemographic and clinical variables were measured or collected. Variables relevant to the present study were the body mass index, BMI, and the markers of kidney function/dysfunction, namely serum creatinine and the estimated glomerular filtration rate, eGFR [26,27].

### 2.2. Data Analysis

Beta-cell function was assessed by the OGTT through both serum insulin and C-peptide-based indices, as well as through parameters derived by mathematical modeling. From insulin, we calculated the insulinogenic index, IGI, equal to the 30 min to fasting insulin difference divided by the same difference in glucose, and the corresponding index using C-peptide rather than insulin, IGI_CP_ [28,29]. We also calculated other “insulinogenic-like” indices, again with both insulin and C-peptide: the area-under-the-curve (AUC) of insulin during the whole OGTT duration divided by the same AUC of glucose, and the similar index with C-peptide, i.e., AUC_INS_/AUC_GLU_ and AUC_CP_/AUC_GLU_, respectively, as well as the corresponding suprabasal components of those indices, i.e., ΔAUC_INS_/ΔAUC_GLU_ and ΔAUC_CP_/ΔAUC_GLU_ [29]. For a more complete analysis, we also considered indices of beta-cell function at fasting, i.e., the ratio of fasting insulin to glucose. INS_f_/GLU_f_, and that of fasting C-peptide to glucose, CP_f_/GLU_f_.

Beta-cell function was also assessed by a mathematical modeling approach [30,31], which has proven to be effective and reliable [32]. Briefly, in our model approach, insulin secretion is represented as the sum of two components, i.e., S_g_(t) and S_d_(t), where t is time. The first component designates the dependence of insulin secretion on absolute glucose concentration (GLU), and it is characterized by a nonlinear dose-response function, f(GLU). The mean value of the dose-response slope is denoted as glucose sensitivity (GLUSENS) and represents the sensitivity to glucose of the beta-cell insulin secretion; GLUSENS is the most relevant beta-cell function parameter provided by the model. The dose response is modulated by a time-dependent potentiation factor, P(t); thus, S_g_(t) = P(t)∙f(GLU). The ratio of the potentiation factor at the end of the OGTT to that at zero minutes is denoted as PFR (potentiation factor ratio). The second insulin secretion component, S_d_(t), represents a dynamic dependence of insulin secretion on the rate of change of glucose concentration and it is termed derivative component. S_d_(t) is proportional to the glucose time derivative (when the glucose derivative is positive, otherwise S_d_(t) = 0); the proportionality constant is denoted as rate sensitivity (RSENS). In addition to GLUSENS, RSENS and PFR, other parameters of interest are the insulin secretion rates at prescribed glucose values. Typical values that are considered are 5, 6, 7 mmol/L of glucose, the parameters being named as ISR_5_, ISR_6_, ISR_7_, respectively.

We also assessed insulin resistance and sensitivity indices. We calculated insulin resistance at fasting through HOMA-IR [33,34] and insulin sensitivity during the OGTT through PREDIM [35,36]. 

Furthermore, we introduced a new index in this study that we named COSUGI (Combined Suprabasal Glucose and Insulin) with the intended use as an alpha-cell function surrogate marker. Insulin and glucose are the main drivers in the suppression of glucagon after glucose administration, and COSUGI was defined as (ΔAUC_GLUnorm_ + ΔAUC_INSnorm_)/2. ΔAUC_GLUnorm_ is defined as the ΔAUC_GLU_ value in a single patient normalized to the average ΔAUC_GLU_ calculated over all patients. A similar definition holds for ΔAUC_INSnorm_. We calculated a similar index using C-peptide rather than insulin. Thus, to distinguish the two index versions, we named them COSUGI_INS_ and COSUGI_CP_, which were based on insulin and on C-peptide, respectively. Such indices may be surrogate markers of alpha-cell function.

Both indices and model-based parameters were calculated with MATLAB version R2020a (The MathWorks^®^, Natick, MA, USA).

### 2.3. Statistical Analysis

Differences between patient groups were tested by analysis of variance (ANOVA), with adjustment for possibly confounding variables by general linear models. Specifically, we tested for possible differences in the analyzed variables between the group of patients who developed PTDM and that of those patients who remained diabetes-free (NON-PTDM). In addition, for a subset of the variables, we tested for possible differences between obese and nonobese (OB, NON-OB), elderly and nonelderly (ELDER, NON-ELDER), and male and female (M, F) patient groups. Obesity was defined as BMI > 27 kg/m^2^, whereas the definition of elderly was based on the calculation of the median age in the whole study population. We considered HOMA-IR, PREDIM, BMI, age and treatment arm (basal insulin intervention or standard-of-care) as variables for adjustments.

A possible relationship between variables was assessed by linear regression analysis. In order to perform verifications on the reliability of our modeling approach for beta-cell function assessment, regression analysis was performed among GLUSENS (the most relevant model-based parameter) and ΔAUC_CP_/ΔAUC_GLU_ (the non-model-based index more similar to GLUSENS, as known from previous analyses in other patient’s populations). Since C-peptide clearance is influenced by kidney function and the calculation of GLUSENS relies on the assumption of C-peptide kinetics, we assessed if the model-based approach would introduce bias, on top of a possible increase of overall C-peptide during the OGTT with declining kidney function. 

Therefore, we considered the residuals of the regression analysis between GLUSENS and ΔAUC_CP_/ΔAUC_GLU_ (residuals being a measure of the case-by-case difference between the two variables), and hence, we performed a second regression analysis: those residuals vs. an index of kidney function (we tested both creatinine and eGFR). Indeed, a significant relationship between those residuals and either creatinine or eGFR would indicate that the difference between GLUSENS and ΔAUC_CP_/ΔAUC_GLU_ depends upon the degree of kidney function/dysfunction. In contrast, the absence of that relationship would indicate no such dependence, and this information is of relevance to conclude whether our modeling approach can be reliably applied to the particular patients (kidney transplant recipients) of the present study.

Before performing the above indicated statistical tests, we analyzed the variable distributions and performed natural logarithm transformation in case of skewed distributions. Two-sided *p*-values less than 0.05 were considered statistically significant. This was an exploratory analysis, and therefore, no adjustment for multiple testing was performed. Data are reported as mean ± standard deviation (SD) unless otherwise specified. The indicated statistical analyses were performed in R (The R Foundation, version 3.6.3) and contributing packages.

## 3. Results

### 3.1. Basic Characteristics in Patients with PTDM and without PTDM

Among 263 participants included in the ITP-NODAT study, 20 patients discontinued the study before month 6. A group of 191 patients with sufficient data at the 6 months OGTT were studied in the present investigation, of which 26 had PTDM. The basic patient characteristics at baseline (i.e., immediately before transplantation) are reported in Table 1. Men and women proportions were comparable between PTDM and NON-PTDM. Patients with PTDM were older and had higher BMI than patients without PTDM. As expected, fasting glycemia and HbA1c were both higher in PTDM. In contrast, there were no differences between the two groups in the markers of kidney function (i.e., creatinine and eGFR). Table 1 also reports the basic patient characteristics at 6 months. BMI, fasting glycemia and HbA1c were again higher in PTDM than in NON-PTDM, whereas the other variables were not significantly different between the two groups.

As regards the baseline to 6 months changes (Table 1), patients who developed PTDM slightly decreased their BMI. However, HbA1c was higher at 6 months, whereas fasting glucose showed only a tendency to increase (statistical significance not reached). In those who did not develop PTDM, only BMI tended to decrease, whereas both fasting glycemia and HbA1 increased. C-peptide levels markedly decreased in both groups from baseline to the 6 months timepoint (Table 1).

### 3.2. Beta-Cell Function Assessed by Indices Based on Insulin or C-Peptide

Table 2 reports several indices of beta-cell function at 6 months, as derived by serum insulin and C-peptide levels. Beta-cell function at fasting, as represented by both INS_f_/GLU_f_ and CP_f_/GLU_f_, showed a tendency to be higher in PTDM than in NON-PTDM, although statistical significance was not reached. However, in the dynamic conditions of the OGTT, the suprabasal area-under-the-curve of insulin normalized to that of glucose, ΔAUC_INS_/ΔAUC_GLU_ was markedly lower in PTDM, with an average value less than half of that observed in NON-PTDM (see relative difference in Table 2; *p* = 2.97 × 10^−11^). This observation was also seen in the corresponding index based on C-peptide rather than insulin, ΔAUC_CP_/ΔAUC_GLU_ (*p* = 1.95 × 10^−10^). When considering the first 30 min of the OGTT (which may be assumed as first-phase insulin secretion), beta-cell function appeared again impaired, as mirrored by both IGI and its C-peptide-based version, IGI_CP_ (Table 2; *p* = 2.03 × 10^−8^ and *p* = 6.55 × 10^−6^, respectively). Overall, beta-cell function appeared markedly impaired in PTDM, as shown by the two “global” indices, AUC_INS_/AUC_GLU_ and AUC_CP_/AUC_GLU_ (Table 2; *p* = 1.55 × 10^−6^ and *p* = 3.45 × 10^−7^, respectively).

### 3.3. Reliability of Beta-Cell Function Assessment through Modeling Analysis 

**Figure 1:**
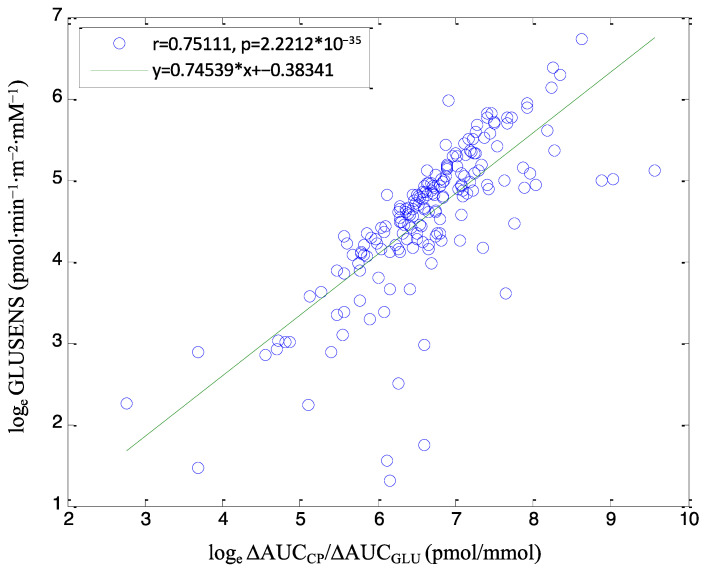
Model-based parameter versus a non-model-based index of beta cell function. Caption: Regression plot between log_e_ of the model-based parameter GLUSENS and log_e_ of the non-model-based index ΔAUC_CP_/ΔAUC_GLU_ in the pooled cohort. Green line: linear regression line. * indicates multiplication.

We performed regression analysis between the model-based parameter called glucose sensitivity (GLUSENS, the most relevant parameter in our modeling approach) and a non-model-based index computed over the whole OGTT duration, ΔAUC_CP_/ΔAUC_GLU_. The latter is, in fact, the non-model-based index closest to the model-based GLUSENS. Figure 1 reports the regression plot of the log_e_ transformation of GLUSENS and ΔAUC_CP_/ΔAUC_GLU_ values in PTDM and NON-PTDM pooled.

Of note, in agreement with the known relationship between ΔAUC_CP_/ΔAUC_GLU_ and GLUSENS in the general population, in our population of renal transplant patients, we also observed a quite high correlation between the two, as mirrored by the highly significant *p*-value (*p* = 2.22 × 10^−35^). On the other hand, the r^2^ parameter was not extremely high (r^2^ = 0.56), indicating that GLUSENS and ΔAUC_CP_/ΔAUC_GLU_ are correlated but not extremely similar and, thus, not interchangeable variables.

**Figure 2:**
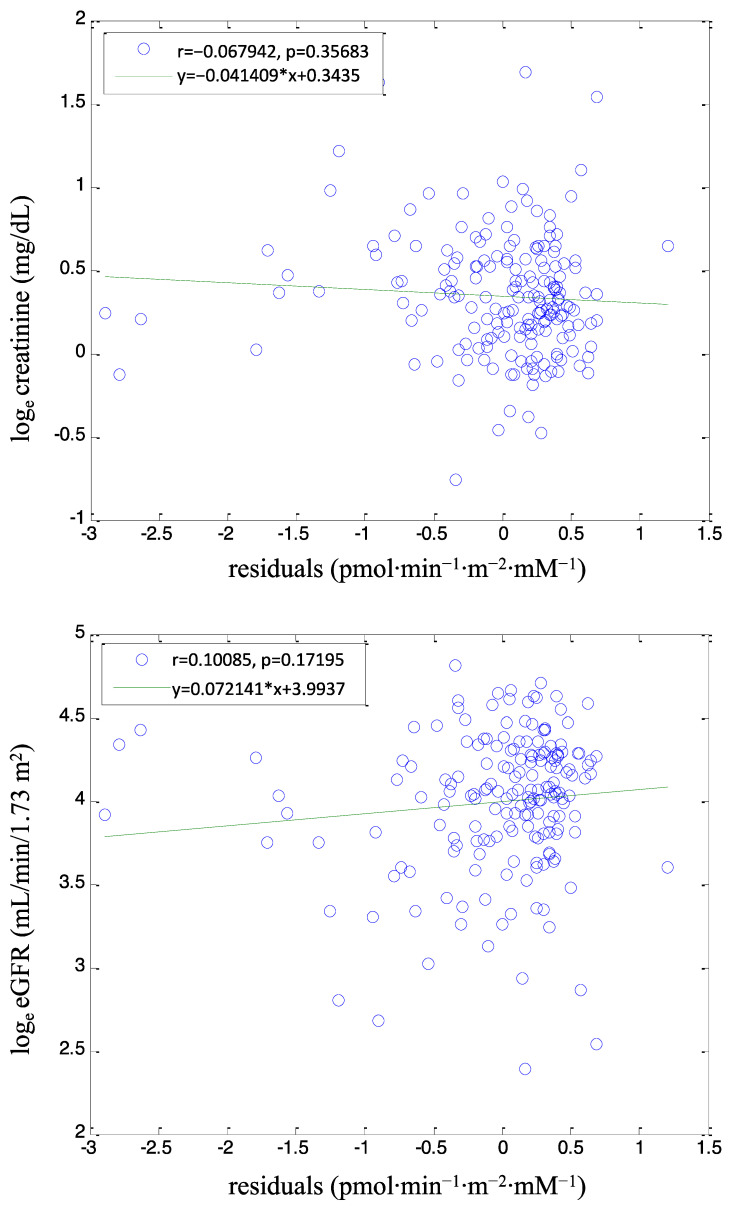
Regression residuals versus parameters of kidney function. Caption: Regression plot between residuals of linear regression (log_e_ GLUSENS vs. log_e_ ΔAUC_CP_/ΔAUC_GLU_) and either log_e_ creatinine (**upper panel**) or log_e_ eGFR (**lower panel**) in the pooled cohort. Green line: linear regression line. * indicates multiplication.

We then analyzed the relationship between the residuals of the ΔAUC_CP_/ΔAUC_GLU_ vs. GLUSENS regression and either creatinine or eGFR (both being markers of renal function/dysfunction). Related plots are reported in Figure 2. For both creatinine and eGFR, there is clearly no significant relationship with the residuals of the GLUSENS vs. ΔAUC_CP_/ΔAUC_GLU_ regression analysis (*p* = 0.36 for creatinine and *p* = 0.17 for eGFR). The lack of relationship between such residuals and two renal dysfunction markers (creatinine and eGFR) allows concluding that the difference between GLUSENS and AUC_CP_/ΔAUC_GLU_ does not depend on the renal dysfunction degree. This concept will be discussed later.

In summary, in the studied subjects, our model-based approach appears not to suffer from possible inaccuracies in C-peptide kinetics assessment. Therefore, the model-based parameters (especially GLUSENS, which is the most important one) can be deemed as reliable. Thus, in the following section, we report the model-based parameter values in the PTDM and NON-PTDM groups.

### 3.4. Beta-Cell Function Assessed by the Model-Based Parameters

**Figure 3:**
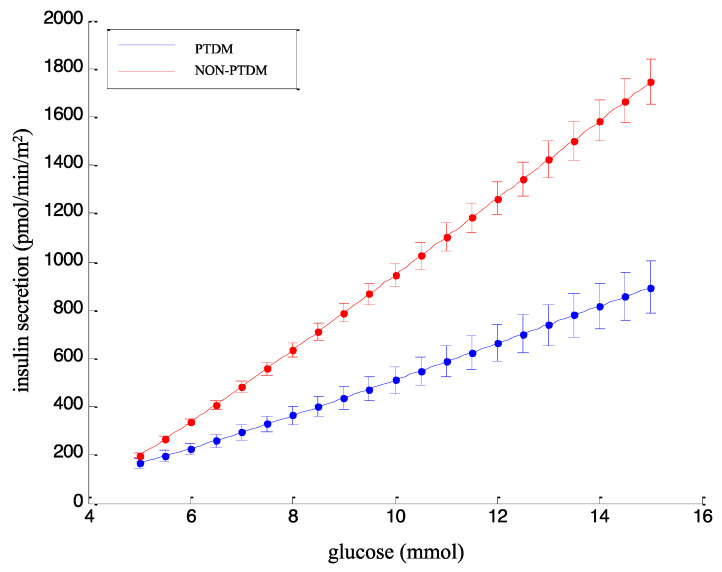
Dose-response plot in PTDM versus NON-PTDM. Caption: Dose-response function in PTDM and NON-PTDM groups. Data are mean ± standard error, SE (SE rather than SD is used here for better figure readability).

In addition to the non-model-based indices, Table 2 reports the model-based beta-cell function parameters. GLUSENS was markedly decreased in PTDM, with the average value being more than 50% lower in patients with PTDM than in patients remaining diabetes-free (Table 2; *p* = 1.46 × 10^−5^), in agreement with the non-model-based indices described above. Figure 3 shows the dose-response function in patients with PTDM and without PTDM, of which the average slope is reported in Table 2 as the GLUSENS parameter. Of note, in PTDM, the dose-response slope was markedly lower than in NON-PTDM.

Regarding the other model-based parameters (Table 2), the rate sensitivity, RSENS, showed a tendency to decrease in PTDM, but statistical significance was missing. The potentiation factor ratio, PFR, was found to be similar in the two groups. Some differences were observed in the series of beta-cell function parameters indicating the insulin secretion rate at specific glucose values. Secretion at 5 mmol/L glucose, ISR_5_, showed only a tendency to be lower in PTDM, whereas secretion at 6 and 7 mmol/L glucose, ISR_6_ and ISR_7_, were markedly lower in PTDM (*p* = 5.44 × 10^−5^ and *p* = 1.59 × 10^−6^, respectively), in agreement with the lower dose-response slope in PTDM (see again Figure 3).

### 3.5. Adjusting the Model-Based Beta-Cell Glucose Sensitivity Parameter in the Comparison between Groups

For the best accuracy in the analysis of the GLUSENS difference between the studied patient groups, it is appropriate to consider adjustment for some possibly relevant variables. First, one should consider possible adjustments for insulin resistance or its reciprocal, i.e., insulin sensitivity. Table 2 reports HOMA-IR (index of insulin resistance at fasting) and PREDIM (index of OGTT-derived insulin sensitivity) in the PTDM and NON-PTDM groups. HOMA-IR tended to be higher in PTDM, but statistically significant difference was not reached. In contrast, PREDIM was clearly significantly decreased in PTDM (*p* = 6.23 × 10^−6^), with a reduction of ~30% as compared to NON-PTDM. Thus, ANOVA of GLUSENS between the two groups was adjusted for PREDIM, and even after such adjustment, the GLUSENS difference between the two groups remained clearly evident (*p* = 0.0013). 

When adjusting for either age or BMI, the difference of GLUSENS between PTDM and NON-PTDM remained markedly significant (*p* = 0.0001 for age adjustment, and *p* = 7.35 × 10^−5^ for BMI adjustment). When adjusting simultaneously for PREDIM, age and BMI, the GLUSENS difference between the two groups remained significant (*p* = 0.0055). 

The proportion of patients undergoing the two treatment arms (standard-of-care versus basal insulin intervention) was similar in the two groups. When adjusting for treatment arm, the GLUSENS difference remained statistically significant (*p* = 1.08 × 10^−5^).

### 3.6. Model-Based Beta-Cell Function in Patients Stratified According to Different Criteria

When stratifying the patients into an obese group (OB: BMI > 27 kg/m^2^, *n* = 65) and nonobese group (NON-OB: BMI ≤ 27 kg/m^2^, *n* = 126), there was no significant difference in GLUSENS or in ISR_5,6,7_. RSENS tended to be higher in NON-OB, but significance was not reached (Table 3). However, a significant difference was observed in the potentiation parameter, PFR (*p* = 0.0046). In the comparison between the elderly group (ELDER: age > 52.5 years, *n* = 89) and nonelderly group (NON-ELDER: age ≤ 52.5 years, *n* = 102), we found that GLUSENS was lower in ELDER than in NON-ELDER (*p* = 0.0056). ISR_7_ was also slightly lower (*p* = 0.0263). In contrast, the other parameters were similar in the two groups. When comparing males (M, *n* = 122) and females (F, *n* = 69), we did not find any parameter significantly different between the two groups. RSENS showed a tendency to be higher in F, but without statistical significance.

### 3.7. The COSUGI Index

**Figure 4:**
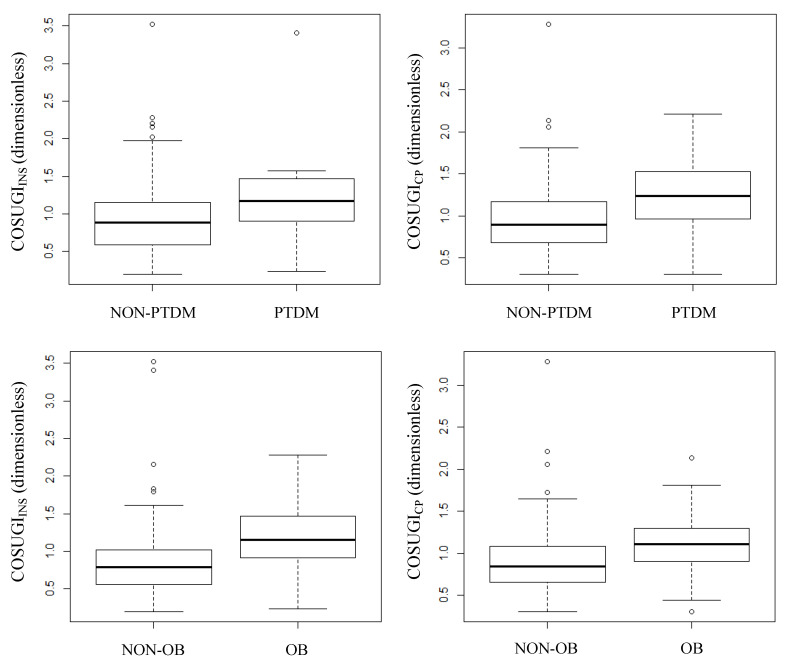
COSUGI index stratified according to different criteria. Caption: Box plots of COSUGI_INS_ and COSUGI_CP_ in PTDM and NON-PTDM groups (**upper panels**, left to right) and in OB and NON-OB groups (**lower panels**, left to right).

We have defined a new index based on the average between the normalized AUC of insulin (or C-peptide) and glucose. Both COSUGI index versions (COSUGI_INS_ and COSUGI_CP_) were different between PTDM and NON-PTDM, being higher in the former (see Table 2; *p* = 0.0168 for COSUGI_INS_ and *p* = 0.0019 for COSUGI_CP_). Figure 4 reports the box plot of the two COSUGI index versions in PTDM and NON-PTDM. When considering the patients stratified according to the other investigated criteria, we found a significant difference between OB and NON-OB: 1.18 ± 0.48 vs. 0.88 ± 0.56 (*p* = 1.26 × 10^−5^) for COSUGI_INS_, 1.11 ± 0.32 vs. 0.93 ± 0.45 (*p* = 0.0006) for COSUGI_CP_, in OB vs. NON-OB (see box plots in Figure 4).

## 4. Discussion

In this study, we analyzed the glucometabolic condition of kidney transplant recipients with or without PTDM at 6 months after transplantation, with a special focus on beta-cell function. To our knowledge, this study is the first to compare the glucometabolic condition based on both traditional indices and model-based analysis between patients with PTDM and without PTDM.

The most relevant finding of our analysis is that patients with PTDM had remarkably impaired beta-cell function as compared to patients without PTDM, being more than 50% lower in PTDM than in NON-PTDM, according to different beta-cell function variables. Of note, both model-based parameters (the GLUSENS parameter) and non-model-based indices (ΔAUC_INS_/ΔAUC_GLU_ and ΔAUC_CP_/ΔAUC_GLU_) consistently agreed in showing such level of difference in beta-cell function between the two groups. With regard to insulin sensitivity (the other typical main driver of glucose tolerance), we also found a clear impairment in PTDM, but it appeared somehow less marked than the beta-cell function impairment (about 30% lower in PTDM).

Beta-cell function at fasting, as represented by both INS_f_/GLU_f_ and CP_f_/GLU_f_, showed a tendency to be higher in PTDM than in NON-PTDM. This observation suggests an attempt of the beta cell to counteract the hyperglycemic condition. However, in the dynamic conditions of the OGTT, the failure of the beta cell in counteracting hyperglycemia clearly emerged. Indeed, patients with PTDM had ΔAUC_INS_/ΔAUC_GLU_ on average half of the NON-PTDM group. When considering the first 30 min of the OGTT (which may be assumed as first-phase insulin secretion), the beta-cell function appeared again impaired, as mirrored by both IGI. This observation appeared consistent with the notion of marked impairment in first-phase beta-cell function in T2DM when assessed in the first 8–10 min of an intravenous glucose tolerance test. In agreement with the known relationship between ΔAUC_CP_/ΔAUC_GLU_ and GLUSENS in the general population, we also observed quite a strict correlation (r^2^ = 0.56, *p* = 2.22 × 10^−35^) between the two in our population of kidney transplant recipients, indicating that these variables were closely associated.

Our results suggest, from a mechanistic point of view, that early insulin administration posttransplant can be helpful in avoiding beta-cell exhaustion and, hence, preserve residual beta-cell function. Our previous trial [23] showed that protocol adherence could not always be achieved, and treatment adherence is relevant in patients with T2DM on basal insulin therapy [37]. As the most promising effect of insulin treatment in kidney transplant recipients was found in the high-risk population of our previous trial, future studies could consider the opportunity to develop appropriate methods for identifying patients who need particularly robust therapy for the beta-cell. Patients progressing to PTDM also had a clear impairment in insulin sensitivity, compared to patients in the NON-PTDM group, consistent with the concept of diabetes progression, demanding personalized, precisely tailored therapies [38].

In this study, we calculated both model-based parameters and non-model-based indices. Some investigators may wonder whether model-based approaches may disclose information not provided by the simpler non-model-based approaches. In our experience, the parameters based on mathematical models are often less prone to the typical limitations of the simple indices, such as the presence of outliers. Thus, model-based parameters often show a higher ability to disclose subtle differences among groups, possibly in agreement with reference parameters obtained by complex experiments [39,40,41,42]. However, it has to be acknowledged that, in the present study, relevant differences in beta-cell function between the study groups were already clearly shown by some of the non-model-based indices. Nonetheless, we hypothesized that the calculation of the model-based parameters may have corroborated the analysis performed with the non-model-based indices, and this is, in fact, what we have observed since we found good agreement between model and non-model-based variables, at least for the most relevant ones (specifically, GLUSENS parameters and ΔAUC_INS_/ΔAUC_GLU_ as well as ΔAUC_CP_/ΔAUC_GLU_ indices).

On the other hand, in this study, the use of our model-based approach for beta-cell function [30] needed some preliminary verifications. The reason is that our modeling approach requires the calculation of the individual C-peptide kinetics, and this is obtained with a widely used method by Van Cauter et al. [43], which allows C-peptide kinetics assessment from basic variables (sex, age, body weight and height, and knowledge of diabetic or nondiabetic status). Unfortunately, Van Cauter’s study did not include patients with kidney dysfunction. Thus, since some degree of kidney dysfunction is often present following kidney transplantation and C-peptide is mainly cleared by kidneys [44,45,46,47,48,49], we wondered whether we could reliably apply our beta-cell function model, based on the Van Cauter’s approach for C-peptide kinetics, in the context of the present study. To this purpose, we performed regression analysis between our GLUSENS parameter (model-based, thus possibly affected by inaccuracies in the C-peptide kinetics assessment) and the ΔAUC_CP_/ΔAUC_GLU_ index (non-model-based, thus may be influenced by possible C-peptide kinetics alterations due to renal dysfunction but not affected by model-specific inaccuracies). In fact, it is known from previous analyses in different populations that ΔAUC_CP_/ΔAUC_GLU_ is typically “similar” to GLUSENS. We then considered the residuals of such regression analysis, because high residuals mean large differences between GLUSENS and ΔAUC_CP_/ΔAUC_GLU_. The next step was, therefore, to perform another regression analysis between those residuals and one variable representing kidney function (we considered both creatinine and eGFR). In keeping with the hypothesis that our modeling approach (thus, GLUSENS parameter) was affected by the C-peptide kinetics issue, the problem would have been more evident in patients with a higher degree of kidney dysfunction. This would translate into a significant relationship between the GLUSENS and ΔAUC_CP_/ΔAUC_GLU_ residuals (i.e., the degree of difference between them) and the degree of kidney dysfunction (creatinine and eGFR values). Such a relationship was not present at all, this being a clue that there was no additional relevant C-peptide kinetics issue possibly affecting the model approach reliability. On the other side, this appears consistent with the fact that kidney function, as evaluated by eGFR, was stable in most patients in our study cohort.

With our model-based approach, we further examined beta-cell function in our patients by stratifying them according to criteria different from those of glucose tolerance. It has to be acknowledged that sometimes the parameter differences between groups were less frequent than one may expect. However, it must be considered that the parameters of our model approach represent clearly distinct physiological aspects of beta-cell function (this being one of the strengths of our approach) [32,50], and it was already observed in the general population (i.e., nontransplant patients) that only a subset of the various parameters display differences among the studied groups [51].

In our study, we also defined and computed a new index, which we named COSUGI, calculated as the average normalized area-under-the curve of insulin (or C-peptide) plus that of glucose. Interestingly, we found this index to be different (in both the insulin-based and C-peptide-based versions) between PTDM and NON-PTDM. We proposed this index in the hypothesis that this may be a surrogate marker of alpha-cell function (another relevant determinant of glucose homeostasis [52]), although this, of course, cannot be proved with the present data and hence needs investigation in future studies. It has to be acknowledged that the formulation of the prosed index resembles that of an insulin resistance index. However, when performing regression analysis of the index with HOMA-IR or PREDIM, we found a significant (*p* < 0.05) but weak relationship, suggesting that such an index may represent physiological phenomena certainly different from the insulin action. We hypothesize that the index may be somehow related to the alpha-cell function. This is due to the reason that, although several factors may affect alpha-cell function (including environmental ones [53]), the main drivers in the suppression of glucagon (secreted by the alpha cells), following glucose administration are likely insulin and glucose (the higher the insulin and glucose levels, the higher the glucagon suppression propensity) [54,55,56,57]. From these considerations, we derived our hypothesis of the index as an alpha-cell function surrogate marker, to be investigated with appropriate data. 

Opportunities for comparison of our results on beta-cell function with those of previous findings are limited. In a pioneering study by Ekstrand et al. [58], reduced insulin secretion was found in patients with PTDM compared to patients without PTDM with the hyperglycemic clamp. In the study by Midtvedt et al. [59], insulin release in an OGTT was reduced in patients with PTDM compared to patients without PTDM. However, actual beta-cell function indices were not computed since only insulin release data (i.e., without normalization to the glucose levels) was reported. Similar limitations hold for the study by Nam et al. [18], as well as for the study by Zelle et al. [19], where C-peptide levels were assumed as markers of beta-cell function, but again with lack of normalization to the glucose levels, and in addition, only values at fasting were collected. In the study by Hagen et al. [60], both indices of insulin secretion and actual beta-cell function were computed from an OGTT. At baseline, no difference was found between the patients with and without PTDM, whereas at follow-up, the difference between groups was not reported (the study focused on the follow-up to basal differences in each of the two groups). At any rate, we can claim that the findings of the previous studies, though limited and with remarkable methodological and clinical differences from our study, essentially do not show disagreement with our results. 

While the COSUGI index needs further investigation in appropriate patient groups, it has been suggested that hyperglucagonemia may be another contributor to PTDM by Halden et al. [61]. The performed clamp study showed that patients with PTDM had lower insulin secretion and a smaller drop of glucagon release versus patients without PTDM. The maximal suppression from baseline was 43% in PTDM vs. 65% in diabetes-free patients. Therefore, reduced glucose-induced insulin secretion and reduced glucagon suppression may contribute to PTDM in kidney transplant recipients.

In conclusion, in the present study, we have performed a deep appraisal of the beta-cell function, as well as of other glucometabolic variables, in kidney transplant recipients with and without PTDM at 6 months following transplantation. We have found that beta-cell function is markedly impaired in PTDM, although defects in insulin sensitivity are present as well. These findings suggest that early treatment of the beta-cell may be even more robust in those patients with a particularly high risk for PTDM, although also the aspect of insulin resistance should not be totally neglected.

## Figures and Tables

**Table 1:** Patients’ characteristics.

	PTDM	NON-PTDM
Baseline		
Sex (Male/Female)	15/11	107/58
Age (years)	59.88 ± 11.22	48.24 ± 14.64 *
BMI (kg/m^2^)	29.79 ± 6.48	25.14 ± 4.75 *
Fasting plasma glucose (mg/dL)	103.52 ± 25.75	91.89 ± 16.31 *
Fasting serum insulin (µU/mL)	23.30 ± 27.23	14.09 ± 16.44
Fasting serum C-peptide (ng/mL)	12.67 ± 9.48	9.19 ± 5.01 *
HbA1c (%)	5.45 ± 0.46	5.10 ± 0.51 *
Creatinine (mg/dL)	7.52 ± 3.72	7.67 ± 2.70
eGFR (mL/min/1.73 m^2^)	8.23 ± 3.42	8.64 ± 4.11
6-month follow-up		
BMI (kg/m^2^)	28.53 ± 6.32 ^†^	24.87 ± 4.88 *
Fasting plasma glucose (mg/dL)	109.42 ± 21.26	95.85 ± 12.46 *^,†^
Fasting serum insulin (µU/mL)	13.50 ± 9.84	10.28 ± 7.19 ^†^
Fasting serum C-peptide (ng/mL)	4.47 ± 2.75 ^†^	3.63 ± 1.67 ^†^
HbA1c (%)	6.03 ± 0.76 ^†^	5.45 ± 0.51 *^,†^
Creatinine (mg/dL)	1.50 ± 0.46 ^†^	1.51 ± 0.64 ^†^
eGFR (mL/min/1.73 m^2^)	51.86 ± 15.55 ^†^	59.73 ± 21.97 ^†^

Note: Basic characteristics of the patients at baseline (before transplantation) and at 6 months follow-up. Patients are stratified according to their glucose tolerance at 6 months (PTDM: posttransplant diabetes mellitus; NON-PTDM: no occurrence of PTDM). Data are mean ± SD. * *p* < 0.05 in PTDM vs. NON-PTDM; ^†^ *p* < 0.05 baseline vs. 6 months follow-up; BMI: body mass index; HbA1c: glycated hemoglobin; eGFR: estimated glomerular filtration rate.

**Table 2:** Beta-cell function derived by basic indices and by mathematical modeling.

	PTDM	NON-PTDM	PTDM–NON-PTDM Relative Difference (%)
Beta-cell function indices from insulin			
INS_f_/GLU_f_ (pmol/mmol)	14.18 ± 12.24	11.49 ± 7.45	23.40
IGI (pmol/mmol)	44.78 ± 47.47	118.98 ± 114.07 *	−62.37
AUC_INS_/AUC_GLU_ (pmol/mmol)	27.33 ± 26.06	42.74 ± 25.82 *	−36.05
ΔAUC_INS_/ΔAUC_GLU_ (pmol/mmol)	55.18 ± 65.98	163.51 ± 200.13 *	−66.25
Beta-cell function indices from C-peptide			
CP_f_/GLU_f_ (pmol/mmol)	244.09 ± 139.97	224.87 ± 91.71	8.54
IGI_CP_ (pmol/mmol)	288.04 ± 264.49	581.53 ± 468.72 *	−50.47
AUC_CP_/AUC_GLU_ (pmol/mmol)	297.30 ± 159.14	414.98 ± 152.09 *	−28.36
ΔAUC_CP_/ΔAUC_GLU_ (pmol/mmol)	464.96 ± 547.23	1250.28 ± 1519.59 *	−62.81
Beta-cell function parameters from modeling analysis			
GLUSENS (pmol∙min^−1^∙m^−2^∙mM^−1^)	68.44 ± 57.82	143.73 ± 112.91 *	−52.38
RSENS (pmol∙m^−2^∙mM^−1^)	485.87 ± 881.21	941.02 ± 1664.61	−48.37
PFR (dimensionless)	1.35 ± 0.56	1.57 ± 0.90	−13.96
ISR_5_ (pmol∙min^−1^∙m^−2^)	165.01 ± 128.14	196.99 ± 103.28	−16.24
ISR_6_ (pmol∙min^−1^∙m^−2^)	226.51 ± 145.17	331.37 ± 172.25 *	−31.64
ISR_7_ (pmol∙min^−1^∙m^−2^)	293.19 ± 181.12	475.58 ± 273.22 *	−38.35
Other glucometabolic indices			
HOMA-IR (dimensionless)	3.61 ± 2.70	2.49 ± 1.93	44.66
PREDIM (mg/min/kg)	3.65 ± 1.68	5.46 ± 2.57 *	−33.12
COSUGI_INS_ (dimensionless)	1.21 ± 0.56	0.95 ± 0.51 *	27.76
COSUGI_CP_ (dimensionless)	1.23 ± 0.41	0.95 ± 0.39 *	29.29

Note: Beta-cell function indices based on insulin or C-peptide measurements and beta-cell function parameters derived by mathematical modeling. Other glucometabolic indices are also reported. Indices and parameters are calculated from the measurement of plasma glucose, serum insulin and serum C-peptide derived by the 6 months oral glucose tolerance test. Patients are stratified according to their glucose tolerance at 6 months (PTDM: posttransplant diabetes mellitus; NON-PTDM: no occurrence of PTDM). Data in PTDM and NON-PTDM are mean ± SD. The relative difference between PTDM and NON-PTDM was computed as the difference of the average in PTDM versus NON-PTDM normalized to the NON-PTDM value and multiplied by 100. * *p* < 0.05 in PTDM vs. NON-PTDM; GLU: glucose; INS: insulin; CP: C-peptide; f: fasting; IGI: insulinogenic index; IGI_CP_: insulinogenic index from C-peptide; AUC: area-under-the-curve; ΔAUC: suprabasal AUC; GLUSENS: glucose sensitivity; RSENS: rate sensitivity; PFR: potentiation factor ratio; ISR_5,6,7_: insulin secretion rate at 5, 6, 7 mmol/L glucose, respectively; HOMA-IR: homeostasis model assessment–insulin resistance; PREDIM: predicted M value (M: insulin sensitivity from the clamp); COSUGI_INS,CP_: combined suprabasal glucose-insulin index from insulin, C-peptide, respectively.

**Table 3:** Beta-cell function parameters stratified according to different criteria.

	OB	NON-OB	ELDER	NON-ELDER	M	F
Beta-cell function parameters from modeling analysis						
GLUSENS (pmol∙min^−1^∙m^−2^∙mM^−1^)	120.50 ± 116.34	140.18 ± 106.64	109.79 ± 84.72	154.16 ± 125.03 *	138.35 ± 106.04	123.54 ± 117.95
RSENS (pmol∙m^−2^∙mM^−1^)	459.92 ± 656.91	1095.29 ± 1862.34	868.25 ± 1779.15	888.50 ± 1406.86	788.89 ± 1568.99	1039.76 ± 1638.98
PFR (dimensionless)	1.31 ± 0.48	1.66 ± 0.90 *	1.60 ± 0.94	1.49 ± 0.71	1.54 ± 0.88	1.54 ± 0.75
ISR_5_ (pmol∙min^−1^∙m^−2^)	195.66 ± 117.63	191.08 ± 101.70	199.04 ± 114.06	187.06 ± 101.00	188.20 ± 110.23	201.50 ± 103.09
ISR_6_ (pmol∙min^−1^∙m^−2^)	303.37 ± 180.92	324.18 ± 167.93	300.02 ± 156.42	332.01 ± 184.52	317.68 ± 169.21	316.77 ± 181.17
ISR_7_ (pmol∙min^−1^∙m^−2^)	420.63 ± 278.31	466.29 ± 264.80	408.78 ± 226.51	487.38 ± 298.54 *	456.83 ± 262.55	439.58 ± 286.66

Note: Beta-cell function parameters derived by mathematical modeling, calculated from the measurement of plasma glucose, serum insulin and serum C-peptide derived by the 6 months oral glucose tolerance test. Patients are stratified according to different criteria registered at 6 months OB (obese) and NON-OB (nonobese); ELDER (elderly) and NON-ELDER (nonelderly); M (male) and F (female). Data are mean ± SD. * *p* < 0.05 in each pair of groups comparison (OB vs. NON-OB; ELDER vs. NON-ELDER; M vs. F); GLUSENS: glucose sensitivity; RSENS: rate sensitivity; PFR: potentiation factor ratio; ISR_5,6,7_: insulin secretion rate at 5, 6, 7 mmol/L glucose, respectively.

## Data Availability

The data presented in this study are available on request from the corresponding author. The data are not publicly available due to restrictions (privacy/ethical).
